# LncRNA-DANCR Interferes With miR-125b-5p/HK2 Axis to Desensitize Colon Cancer Cells to Cisplatin vis Activating Anaerobic Glycolysis

**DOI:** 10.3389/fonc.2020.01034

**Published:** 2020-07-17

**Authors:** Huijuan Shi, Kejun Li, Jinxin Feng, Gaojie Liu, Yanlin Feng, Xiangliang Zhang

**Affiliations:** ^1^Department of Pathology, The First Affiliated Hospital of Sun Yat-sen University, Guangzhou, China; ^2^Department of Abdominal Surgery, Affiliated Cancer Hospital & Institute of Guangzhou Medical University, Guangzhou, China

**Keywords:** lncRNA-DANCR, colon cancer, microRNA-125b-5p, hexokinase 2, cisplatin resistance, glycolysis

## Abstract

Colon cancer is one of the most prevalent malignancies that lead to high occurrence of cancer-related deaths. Currently, chemotherapies and radiotherapies remain the primary treatments for advanced colon cancer. Despite the initial effectiveness, a fraction of colon cancer patients developed cisplatin resistance, resulting in therapeutic failure. The long non-coding RNA differentiation antagonizing non-coding RNA (DANCR) has been shown to be upregulated in multiple cancers, indicating an oncogenic role of DANCR. This study aims to elucidate the roles of DANCR in regulating cisplatin (CDDP) resistance of colon cancer. We found DANCR was significantly upregulated in colon cancer tissues and cells compared with normal colon tissues and cells. DANCR was upregulated in cisplatin-resistant colon cancer cells. Moreover, overexpression of DANCR significantly desensitized colon cancer cells to cisplatin. On the other way, silencing DANCR dramatically overrode CDDP resistance of colon cancer cells. Bioinformatics prediction revealed DANCR could bind to seeding region of miR-125b-5p as a competitive endogenous RNA. This interference was further validated by luciferase assay. Moreover, we detected a negative correlation between DANCR and miR-125b-5p in colon cancer patient tissues: miR-125b-5p was clearly downregulated in colon cancer tissues and cells. Overexpression of miR-125b-5p significantly sensitized cisplatin-resistant cells. Interestingly, we observed the cisplatin-resistant cells were associated with a significantly increased glycolysis rate. We further identified glycolysis enzyme, hexokinase 2 (HK2), as a direct target of miR-125b-5p in colon cancer cells. Rescue experiments showed overexpression of miR-125b-5p suppressed cellular glycolysis rate and increased cisplatin sensitivity through direct targeting the 3′ UTR of HK2. Importantly, silencing endogenous DANCR significantly induced the miR-125b-5p/HK2 axis, resulting in suppression of the glycolysis rate and increase in cisplatin sensitivity of colon cancer cell. Expectedly, these processes could be further rescued by inhibiting miR-125b-5p in the DANCR-silenced cells. Finally, we validated the DANCR-promoted cisplatin resistance via the miR-125b-5p/HK2 axis from an *in vivo* xenograft mice model. In summary, our study reveals a new mechanism of the DANCR-promoted cisplatin resistance, presenting the lncRNA-DANCR–miR-125b-5p/HK2 axis as a potential target for treating chemoresistant colon cancer.

## Introduction

Colorectal cancer is a commonly diagnosed cancer with poor prognosis and high mortality rate, presenting it as one of the major causes of the cancer-related death worldwide (1, 2). Currently, surgical resection is the major and traditional therapy for colorectal cancer (2). In addition, chemotherapy and radiotherapy are promising approaches for patients with late-stage or metastatic colorectal cancer (3). Cisplatin (CDDP), with the capacity to induce DNA damage, is a widely used chemotherapeutic agent for effective treatments of various types of cancers (4). Despite the initial effectiveness in colon cancer chemotherapy, a fraction of patients developed resistance to CDDP treatment, resulting in therapeutic failure (5). Seriously, because of acquired chemoresistance, the morbidity and mortality of colorectal cancer still remain considerable (5, 6). Therefore, investigating the molecular mechanisms underlying the drug resistance of colon cancer will contribute to improving the clinical outcomes of affected patients.

The long-non-coding RNAs (lncRNA) are a class of RNAs (>200 nt and non-coding) that lack an initiation codon and a termination codon (7–9). Accumulating studies revealed that lncRNAs were important regulators involved in diverse carcinogenesis and progression (7). Currently, lncRNAs are emerging as novel cancer biomarkers for diagnosis and treatment of cancers (10, 11). Thus, exploration of the roles and mechanisms of lncRNAs is an attractive task to develop anticancer agents for overcoming drug resistance. The differentiation antagonizing non-coding RNA (DANCR) was found to be upregulated in a variety of cancers, including colorectal cancer (12), liver cancer (13), breast cancer (14), and prostate cancer (15), presenting it as a cancer diagnostic and prognostic marker. MicroRNAs (miRNAs), which play important roles in multiple cancer progressions, are a class of short, endogenous non-coding RNAs (~20–22 nt) (16). Previous studies reported miR-125b-5p was downregulated and enhanced chemotherapy sensitivity in gallbladder cancer (17), suggesting a suppressive role in chemoresistance. Evidence has suggested that lncRNA could act as miRNA sponges [competitive endogenous RNA (ceRNA)] to downregulate downstream target miRNAs expressions, resulting in derepressing the mRNA targets of miRNAs (18–20). It has been reported DANCR could directly bind miR-216a in hepatocellular carcinoma (21). In addition, DANCR was known to promote progressions of bladder cancer cells by regulating the miR-149/MSI2 axis as a ceRNA (22), suggesting DANCR could be a therapeutic target against colon cancer. However, the precise target miRNAs of DANCR in colon cancer are still under investigation.

Otto H. Warburg first illustrated that compared with normal cells, tumor cells display altered glucose metabolism features that they prefer metabolizing glucose into lactate even with sufficient oxygen. This is called “Warburg effect” and recognized as a new hallmark of cancer (23–26). Importantly, the Warburg effect provides a survival advantage tumor cells under multiple chemotherapeutic agents such as 5-fluorouracil (27), cisplatin (28), and taxol (29), leading to the occurrence of chemoresistance. In this study, we assessed the functions of lnc-DANCR in mediating CDDP resistance in colon cancer. The target miRNA of DANCR and potential mechanisms underlying the DANCR-modulated chemoresistance in colon cancer cells were investigated. Our data will potentiate the DANCR interference as a novel antichemoresistance strategy to improve the outcomes of colon cancer.

## Materials and Methods

### Patient Samples

A total of 35 colon cancer patients were evaluated in this study. Colon tumor tissues and their adjacent normal colon epithelial tissues were collected from the Affiliated Cancer Hospital & Institute of Guangzhou Medical University (Guangzhou, China) between January 2015 and December 2017. Tumor samples obtained from surgically were immediately frozen in liquid nitrogen and stored at −80°C. None of the patients received other radiotherapy or chemotherapy before biopsy. This study was approved by the institutional ethical review boards of the Affiliated Cancer Hospital & Institute of Guangzhou Medical University, and written informed consents were obtained from all patients.

### Cell Culture and Reagents

The equations should be inserted in editable format from the equation editor. Five human colon cancer cell lines, HT-29, SW620, HCT116, SW480, and DLD-1, and one normal human colon epithelial cell line, CRL-1790, were obtained from American Type Culture Collection (ATCC, Manassas, VA, USA). Cells were cultured in Dulbecco modified Eagle medium supplemented with 10% heat-inactivated fetal bovine serum (Gibco, Carlsbad, CA, USA), 100 U/mL penicillin G, and 100 μg/mL streptomycin (Thermo Fisher Scientific, Inc., Rockford, IL, USA) at 37°C with a humidified air containing 5% CO_2_. The cisplatin-resistant colon cancer cell line SW480 was established according to previous description (30). Cells were maintained in Dulbecco modified Eagle medium in a 5% CO_2_ incubator at 37°C. The acquired cisplatin-resistant cells were reselected by treating with cisplatin at 30 μM every 2 months. The rabbit anti–hexokinase 2 (HK2) (#2867); rabbit anti-LDHA (#3582), and rabbit anti–β-actin (#4970) antibodies were purchased from Cell Signaling Technology (Danvers, MA, USA). Cisplatin and oxamate were purchased from Sigma–Aldrich (Shanghai, China).

### Transfections of miRNA, shRNA, and Plasmid DNA

Colon cancer cells were seeded in 24-well-plates at a density of 5 × 10^4^ cells/well for overnight before transfection. When cells were achieved 70–80% confluence, transfections were conducted using the Lipofectamine 3000 reagent (Invitrogen, Carlsbad, CA, USA) according to the manufacturer's instructions. Lentiviral vector-based shRNA targeting lncRNA DANCR (5′-GGAGCTAGAGCAGTGACAATG-3′) or control shRNA was purchased from GenePharma Co. (Shanghai, China). The stably shRNA transfected cells were selected by puromycin (2 μg/mL) within cell culture medium. DANCR knockdown efficiency was validated by quantitative reverse transcriptase–polymerase chain reaction (qRT-PCR) in colon cancer cell lines. Hexokinase 2 overexpression vector and control vector were obtained from Origene.com. The DANCR overexpression vector was constructed by inserting full-length DANCR into pcDNA3.1 vector (Thermo Scientific, Inc.). The control miRNA and miR-125b-5p precursor were synthesized by GenePharma Co. The miR-125b-5p antisense (anti–miR-125b-5p) and the scramble negative controls were synthesized from RiboBio (Guangzhou, China). MicroRNAs were transfected at 50 nM. Forty-eight hours after transfection, cells were further analyzed in downstream experiments.

### Prediction of lncRNA-miRNA and miRNA-mRNA Interactions

The prediction of binding sequence between lncRNA DANCR and miR-125b-5p was analyzed by starBase of ENCORI (http://starbase.sysu.edu.cn/) and LncBase Predicted v.2 (http://carolina.imis.athena-innovation.gr/diana_tools/web/index.php?r=lncbasev2%2Findex-predicted). The prediction of binding of miR-125b-5p on HK2 3′ UTR was analyzed from Targetscan (www.Targetscan.org) and starBase of ENCORI (http://starbase.sysu.edu.cn/). The expressions of miR-125b-5p and the negative correlation between miR-125b-5p and HK2 in colon cancer patients were analyzed from starBase of ENCORI (http://starbase.sysu.edu.cn/).

### Measurements of Cellular Glycolysis Rate

The glucose uptake and lactate product were measured using the Glucose Uptake Assay Kit (ab136955) and l-Lactate Assay Kit (ab65331) from Abcam (Shanghai, China) according to the manufacturer's instruction. The relative results were normalized to cell number of each reaction. Assays were performed in triplicate and repeated at least three times.

### RNA Isolation and qRT-PCR

Total RNAs were isolated by TRIzol reagent (Invitrogen) from colon cancer cell lines and frozen colon tumor tissues according to the manufacturer's instruction. The RNA concentration and quality were measured by a NanoDropND-2000spectrophotometer (Thermo Scientific, Inc.). For mRNA and lncRNA detection, cDNA synthesis was performed by the High-Capacity cDNA Reverse Transcription Kit (Thermo Fisher Scientific, Inc.) according to the manufacturer's instructions. Quantitative PCR reactions were conducted using the SYBR Green qPCR Super Mix reagents (Thermo Fisher Scientific, Inc., Waltham, MA, USA) by the CFX96 Touch sequence detection system (Bio-Rad Laboratories, Inc., Hercules, CA, USA). β-Actin was used as an internal control. For miR-125b-5p detection, the cDNA was synthesized using the Taq Man Advanced miRNA cDNA Synthesis Kit (Thermo Fisher Scientific Inc.), and the RT-PCT reactions were performed using the Mir-X™ miRNA qRT-PCR SYBR® kit (Clontech Laboratories, Inc., Mountainview, CA, USA) according to the manufacturer's instructions. Specific primers for miR-125b-5p, lncRNAs, and mRNAs are shown as follows: DANCR: forward, 5′-GCCACTATGTAGAGGGTTTC-3′ and reverse, 5′-ACCTGCGCTAAGAACTGAGG-3′; GLUT1: forward, 5′-CAGTTCGGCTATAACACTGGTG-3′ and reverse, 5′-GCCCCCGACAGAGAAGATG-3′; LDHA: forward, 5′-ATGAAGGACTTGGCGGATGA-3′ and reverse, 5′-ATCTCGCCCTTGAGTTTGTCTT-3′; β-actin: forward, 5′-CTGAGAGGGAAATCGTGCGT-3′ and reverse, 5′-CCACAGGATTCCATACCCAAGA-3′; miR-125b-5p: forward, 5′-TCCCTGAGACCCTAACTTGTGA-3′ and reverse, 5′-AGTCTCAGGGTCCGAGGTATTC-3′; U6: forward, 5′-CTCGCTTCGGCAGCACA-3′ and reverse, 5′-AACGCTTCACGAATTTGCGT-3′. U6 was used as an internal control. The reaction conditions were as follows: 95°C for 3 min, followed by 40 cycles of 95°C for 30 s and 60°C for 30 s. The relative expressions were calculated using the 2^−ΔΔ*CT*^ method.

### Dual-Luciferase Reporter Gene Assay

Luciferase assay was performed according to previous descriptions (13). Briefly, colon cancer cells were seeded in 24-well-plates at a density of 5 × 10^4^ cells/well and cultured for 24 h. Cells were then cotransfected with 50 nM miR-125b-5p or control miRNAs and 50 ng pGL3-reporter luciferase reporter containing 3′ UTR wild-type (WT)–HK2 or mutated (Mut)–HK2 using Lipofectamine 2000 (Thermo Fisher Scientific Inc.). Forty-eight hours after transfection, cells were collected, and luciferase activity was measured using a dual luciferase reporter assay system on a microplate reader (Promega, Madison, WI, USA) according to the manufacturer's instructions. Firefly luciferase activity was normalized to that of the Renilla luciferase. Experiments were performed in triplicate.

### Measurement of Cellular Glycolysis

The cellular glycolysis rate was measured using the Glucose Uptake Colorimetric Assay Kit (MAK083; Sigma–Aldrich) and the l-lactate assay kit (BioVision, Milpitas, CA, USA) according to the manufacturer's instructions. Relative glycolysis rate was calculated from the absorbance of drug-treated cells/the absorbance of untreated cells. Data were normalized by the cell number of each well. Experiments were performed in triplicate and repeated three times.

### Cell Viability Assay

The equations should be inserted in editable format from the equation editor. Cell viability was measured by MTT [3-(4,5-dimethylthiazol-2-yl)-2,5-diphenyltetrazolium bromide] assay according to previous descriptions (31). Briefly, 5 × 10^3^ cells/well were seeded into 96-well–plates. The next day, cell culture medium was aspirated and washed with phosphate-buffered saline (PBS) followed by adding MTT solution at 37°C for 2 h. Samples were incubated with 0.1 mL 10% sodium dodecyl sulfate (SDS) at 37°C for overnight. The optical density value of formazan concentrations was determined at 540 nm. Relative viability was normalized by cell numbers. Experiments were performed in triplicate and repeated three times.

### Western Blotting

Cells were collected and lysed on ice by RIPA lysis buffer (Tris 20 mM, NaCl 150 mM, 1% Triton X-100) containing 1 × protease inhibitor cocktail (Sigma–Aldrich). After 20-min incubation, samples were centrifuged at 10,000 × g at 4°C for 10 min. Protein concentration was measured using the BCA protein assay kit (Beyotime Institute of Biotechnology, Haimen, China). Equal amount protein (30 μg) from each sample was separated by electrophoresis on a 10% SDS–polyacrylamide gel electrophoresis (Bio-Rad Laboratories, Inc.) and transferred onto nitrocellulose membranes (Bio-Rad Laboratories, Inc.). Membranes were blocked in 5% non-fat milk in PBS with Tween for 1 h at room temperature. After washing twice with TBST (0.1% Tween-20), membranes were incubated with primary antibodies at 1:1,000 dilution overnight at 4°C. After complete washing, membranes were incubated with secondary goat anti–rabbit antibodies (dilution: 1:3,000) for 1 h at room temperature. Protein bands were visualized by Millipore ECL Western Blotting Substrate, Billerica, MA, USA using an imaging system. β-Actin was a loading control, and experiments were repeated three times.

### *In vivo* Nude Mouse Models

The equations should be inserted in editable format from the equation editor. The animal protocol was approved by the ethics committee of Guangzhou Medical University and complied with the animal guidelines of the Institutional Animal Care and Use Committee of the Guangzhou Medical University. BALB/C nude mice (5–6 weeks of age) were maintained in the experimental animal center of Guangzhou Medical University in a pathogen-free environment. Mice were divided into four groups: control shRNA without cisplatin, sh DANCR without cisplatin, control shRNA with cisplatin, and sh DANCR with cisplatin groups. SW480/CDDP R cells 5 × 10^6^ with or without stable knockdown DNACR by shRNA in a volume of 100 mL were subcutaneously injected into the mice to establish xenograft models until tumors reached a size of >100 mm^3^. Tumor size was monitored every 3 days. Mice were then treated as follows: PBS control or cisplatin [20 mg/kg intraperitoneal (i.p.), two times per week]. Mice survival was monitored daily. Tumor volumes were calculated as follows: length × width^2^ × 0.5. After 90 days, the mice were euthanized by CO_2_ method. Xenograft tumor tissues were surgically dissected stored at −80°C for downstream analysis.

### Statistical Analysis

Statistical analysis was performed using the GraphPad Prism 7.0 software (GraphPad Software, La Jolla, CA, USA). Experiments were performed in triplicate and repeated three times. Data are presented as mean ± SD. Student *t*-test was applied to compare difference in two groups. One-way analysis of variance was applied for multiple group comparisons, *p* < 0.05 was considered as statistically significant.

## Nomenclature Results

### LncRNA-DANCR Is Upregulated in Colon Cancer and Positively Correlated With Cisplatin Resistance

**Figure 1:**
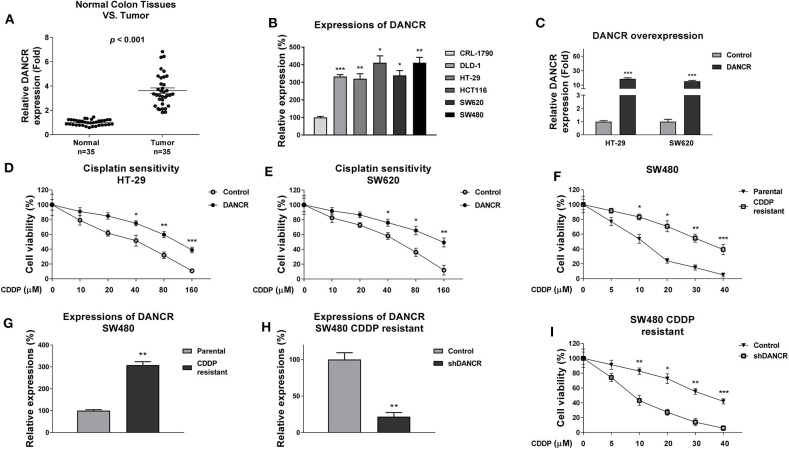
LncRNA DANCR is upregulated in colon cancer and contributes to cisplatin resistance. **(A)** Quantitative RT-PCR results of the DANCR expressions in 35 normal colon tissues and 35 tumor tissues. **(B)** Quantitative RT-PCR results of the DANCR expressions in normal colon epithelial cell and five colon cancer cells. **(C)** DANCR overexpression vector or control vector was transfected into HT-29 and SW620 cells for 48 h. The expressions of DANCR were detected by qRT-PCR. **(D)** HT-29 and **(E)** SW620 cells with control or DANCR overexpression vector transfection were treated with cisplatin at the indicated concentrations for 48 h. Cell viability was measured by MTT assay. **(F)** Establishment of cisplatin-resistant cell line from SW480 cells. The resistance was assessed by treatments with cisplatin at the indicated concentrations. **(G)** The expressions of DANCR were detected from SW480 parental and cisplatin-resistant cells by qRT-PCR. **(H)** SW480/CDDP R cells were transfected with control shRNA or DANCR shRNA for 48 h, the expressions of DANCR were detected by qRT-PCR. **(I)** The above cells were treated with cisplatin at 0, 5, 10, 20, 30, or 40 μM for 48 h; cell viabilities were measured by MTT assay. Data are presented as mean ± SD. *p < 0.05, **p < 0.01, and ***p < 0.001.

Previous studies revealed an oncogenic role of DANCR in multiple cancers (12–15), suggesting DNACR is involved in chemosensitivity. To investigate the functions of DANCR in chemoresistant colon cancer, we first evaluated DANCR expressions in colon tumors and their adjacent normal tissues. As shown in Figure 1A and Figure S1, DANCR expressions were significantly upregulated in 35 colon tumors, consistent with previous reports (12). Moreover, the DANCR expressions were compared in normal colon epithelial cell line, CRL-1790, and five colon cancer cell lines. Expectedly, expressions of DANCR were significantly elevated in colon cancer cells (Figure 1B). Given that DANCR is positively associated with colon cancer, to further assess the potential functions of DANCR in cisplatin sensitivity, we transfected DANCR overexpression lentiviral vector into two colon cancer cell lines, HT-29, and SW620 (Figure 1C). Intriguingly, we observed apparently decreased cisplatin sensitivities of HT-29 and SW620 ells with DNACR overexpression (Figures 1D,E), indicating DNACR contributes to chemoresistance of colon cancer cells. To validate this correlation, we established a cisplatin-resistant colon cancer cell line (SW480/CDDP R) via continuous exposing SW480 cells to cisplatin by gradually increased concentrations of cisplatin. As shown in Figure 1F, the acquired cisplatin-resistant SW480 cells could tolerate higher concentrations of cisplatin. Compared to SW480 parental cells, the IC_50_ of cisplatin-resistant cells increased to 36.7 μM, which was ~3-fold higher than that of parental cells (11.7 μM) (Figure 1F). We detected a significantly increased DANCR level in SW480/CDDP R cells compared with SW480 parental cells (Figure 1G), suggesting that endogenous DANCR positively associates with cisplatin resistance. We thus hypothesized inhibition of endogenous DANCR would sensitize cisplatin-resistant cells. To verify this, we knocked down DANCR in SW480/CDDP R cells (Figure 1H) and examined the responses of cells with low DANCR expression to cisplatin treatments. As we expected, SW480/CDDP R cells with DANCR silencing showed significantly increased sensitivity of cisplatin compared with control cells (Figure 1I). Taken together, the above results consistently demonstrated DNACR played an oncogenic role in colon cancer cells and contributed to cisplatin resistance.

### DANCR Negatively Modulates miR-125b-5p Expression via Sponging It as a ceRNA

**Figure 2:**
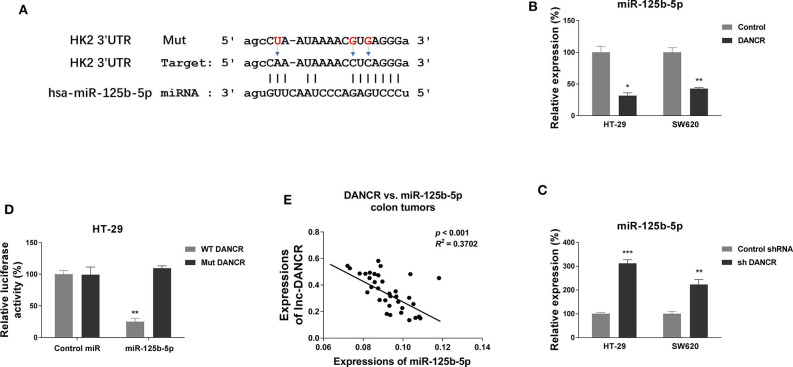
LncRNA DANCR suppresses miR-125b-5p expression via sponging it as a ceRNA. **(A)** Prediction of binding sites of DANCR on miR-125b-5p, as well as the binding sites mutants of DANCR. **(B)** HT-29 and SW620 cells were transfected with control or DANCR overexpression vector for 48 h. The expressions of miR-125b-5p were detected by qRT-PCR. **(C)** HT-29 and SW620 cells were transfected with control shRNA or DANCR shRNA for 48 h. The expressions of miR-125b-5p were detected by qRT-PCR. **(D)** HT-29 cells were cotransfected with luciferase reporter vectors containing WT-DANCR or Mut-DANCR and control miR or miR-125b-5p. The relative luciferase activity was measured by luciferase assay. **(E)** Negative correlation between DANCR and miR-125b-5p expressions was observed from colon cancer tissues. Data are presented as mean ± SD. *p < 0.05, **p < 0.01, and ***p < 0.001.

We continued to explore the molecular mechanisms for the DANCR-promoted cisplatin resistance. Literature research revealed that lncRNAs could serve as sponges to sequester expressions of miRNAs (18–20). We then searched the online software starBase http://starbase.sysu.edu.cn/ and LncBase Predicted v.2 to seek a tumor-suppressive miRNA, which might be the potential target of DANCR. Interestingly, we noticed the seeding region of miR-125b-5p was predicted to complementarily bind with DANCR (Figure 2A). Concomitantly, bioinformatics analysis from the same online software indicated miR-125b-5p was significantly downregulated in colon cancer (Figure S2). We then asked whether DANCR acts as a ceRNA of miR-125b-5p in colon cancer cells. HT-29 and SW620 cells were transfected with DANCR overexpression or knockdown vector. Consistent results demonstrated that colon cancer cells with higher DANCR levels accompanied lower miR-125b-5p expressions, and *vice versa* (Figures 2B,C). To further validate the direct ceRNA binding of DANCR on miR-125b-5p, we constructed luciferase reporter vectors containing original (WT) or nucleotides Mut DANCR (Figure 2A). Luciferase vector was cotransfected with control miRNA or miR-125b-5p into HT-29 cells. Results from luciferase reporter assay clearly illustrated that miR-125b-5p could only bind to WT but not mutant DANCR, resulting in a marked reduction in luciferase activity (Figure 2D). We examined the expressions of DANCR and miR-125b-5p in 35 colon cancer tissues by qRT-PCR, and consistent results demonstrated a significant inverse correlation between DANCR and miR-125b-5p: higher DANCR mRNA levels accompanied with lower miR-125b-5p expressions in colon cancer tissues (Figure 2E and Figure S3). Together, these results implied that DANCR could directly bind to miR-125b-5p and serve as its ceRNA in colon cancer.

### miR-125b-5p Is Negatively Associated With Colon Cancer and Decreases Cisplatin Resistance

**Figure 3:**
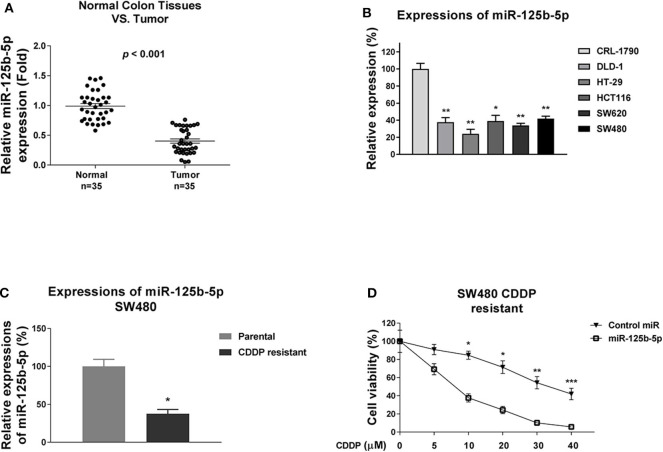
miR-125b-5p is negatively associated with colon cancer and cisplatin resistance. **(A)** Quantitative RT-PCR results of the miR-125b-5p expressions in 35 normal colon tissues and 35 tumor tissues **(B)** Quantitative RT-PCR results of the miR-125b-5p expressions in normal colon epithelial cell and five colon cancer cells. **(C)** Expressions of miR-125b-5p were detected from SW480 parental and cisplatin-resistant cells by qRT-PCT. **(D)** Cisplatin-resistant SW480 cells were transfected with control miR or miR-125b-5p, followed by cisplatin treatments at the indicated concentrations. Cell viability was measured by MTT assay. Data are presented as mean ± SD. *p < 0.05, **p < 0.01, and ***p < 0.001.

The above results revealed DNACR is positively associated with cisplatin resistance and negatively regulates miR-125b-5p. Consequently, we reasoned miR-125b-5p acts as a tumor suppressor in colon cancer and sensitizes colon cancer cells to cisplatin. To validate that, we examined the miR-125b-5p expression in colon tumors and their adjacent normal tissues. Results in Figure 3A and Figure S2 illustrated miR-125b-5p was significantly down-regulated in colon cancers. Furthermore, the miR-125b-5p expressions were detected in normal colon epithelial cell line and five colon cancer cell lines and consistent results showed obviously decreased miR-125b-5p expressions in cancer cells compared with normal cells (Figure 3B). Consequently, we observed attenuation of miR-125b-5p in cisplatin-resistant colon cancer cells (Figure 3C), and exogenous overexpression of miR-125b-5p significantly sensitized colon cancer cells to cisplatin (Figure 3D), indicating miR-125b-5p might be a therapeutic agent for overcoming cisplatin resistance.

### Cisplatin-Resistant Cells Exhibit Elevated Glycolysis Rate

**Figure 4:**
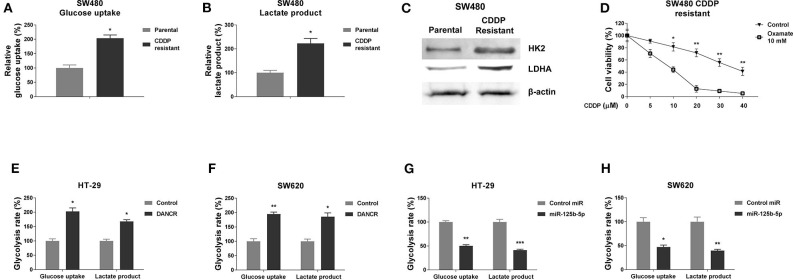
DANCR and miR-125b-5p oppositely regulate glycolysis of gastric cancer cells. **(A)** Glucose uptake and **(B)** lactate product were measured in SW480 parental and cisplatin-resistant cells. **(C)** Protein expressions of HK2, LDHA were detected in SW480 parental, and cisplatin-resistant cells by Western blot. β-Actin was an internal control. **(D)** SW480 cisplatin-resistant cells were cotreated with control or oxamate at 10 mM plus cisplatin at the indicated concentrations for 48 h; the cell viability was measured by MTT assay. **(E)** HT-29 and **(F)** SW620 cells were transfected with control vector or DANCR overexpression vector for 48 h; the glucose uptake and lactate product were measured. **(G)** HT-29 and **(H)** SW620 cells were transfected with control miR or miR-125b-5p precursor for 48 h; the glucose uptake and lactate product were measured. Data are presented as mean ± SD. *p < 0.05, **p < 0.01, and ***p < 0.001.

Accumulating evidence revealed that the dysregulated cellular glycolysis is tightly associated with tumor characteristics, including chemoresistance (26–28). We thus assessed the glucose uptake and lactate production, two glycolysis speed-limit reactions, which are readouts of glycolysis rate. As we expected, the SW480/CDDP R cells exhibited significantly increased glucose uptake (Figure 4A) and lactate product (Figure 4B) rates compared with parental cells, suggesting targeting glycolysis might sensitize colon cancer cells to cisplatin. In addition, we examined the protein expressions of HK2 and LDHA, two enzymes that catalyze the glycolysis key reactions (23, 25, 26). The SW480/CDDP R cells showed clearly upregulated HK2 and LDHA protein expressions by Western blot (Figure 4C). We next tried to block the pyruvate to lactate reaction by treating glycolysis inhibitor, oxamate, followed by the measurements of cisplatin sensitivities. Expectedly, SW480/CDDP R cells showed obviously increased cisplatin sensitivity under oxamate treatment compared with control treatment (Figure 4D). Taken together, these data demonstrated an upregulated cellular glycolysis rate in cisplatin-resistant colon cancer cells.

### DANCR and miR-125b-5p Inversely Regulate Glycolysis Rate of Colon Cancer Cells

Giving the evidence that DANCR and miR-125b-5p exhibited contrary roles in colon cancer and cisplatin resistance, we asked whether they could oppositely regulate glycolysis of colon cancer cells. As shown in Figures 4E,F, overexpression of DANCR in HT-29 and SW620 cells significantly promoted the glucose uptake and lactate product. On the other way, exogenous overexpression of miR-125b-5p clearly suppressed the glycolysis rate of colon cancer cells (Figures 4G,H). These results were consistent with roles of DANCR and miR-125b-5p in colon cancers, respectively.

### miR-125b-5p Directly Targets Glycolysis Enzymes HK2 and Inhibits Cellular Glycolysis

**Figure 5:**
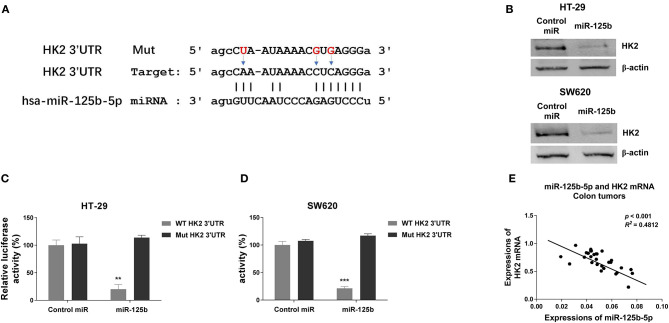
Hexokinase 2 is a direct target of miR-125b-5p in colon cancer. **(A)** The predicted binding sites of miR-125b-5p on 3′ UTR of HK2. **(B)** HT-29 and SW620 cells were transfected with control miR or miR-125b-5p for 48 h. The protein expressions of HK2 were detected by Western blot. **(C)** HT-29 and **(D)** SW620 cells were cotransfected with luciferase reporter vectors containing WT-3′ UTR or mutant 3′ UTR of HK2 and control miR or miR-125b-5p. The relative luciferase activity was measured by luciferase assay. **(E)** A negative correlation between HK2 mRNA and miR-125b-5p expressions was observed from colon cancer tissues. Data are presented as mean ± SD. *p < 0.05, **p < 0.01, and ***p < 0.001.

To further investigate the underlying molecular mechanism by which DANCR and miR-125b-5p act in colon cancer, bioinformatics-based target prediction analysis was performed from TargetScan (http://www.targetscan.org) to seek the potential targets of miR-125b-5p in colon cancer cells. As displayed in Figure 5A, 3′ UTR of HK2, which catalyzes the conversion of glucose to glucose-6-phosphate (G6P), was found to contain binding sequences of miR-125b-5p. To confirm the prediction, we transfected HT-29 and SW620 cells with control or miR-125b-5p precursor, and results from Western blot consistently showed significantly suppressed HK2 protein expressions in miR-125b-5p–overexpressing cells (Figure 5B). Consistent results from the starBase of ENCORI indicated the mRNAs of HK2 in colon cancer tissues were negatively correlated with miR-125b-5p (Figure S4). To validate the direct binding, luciferase reporter assay was further performed by cotransfection of luciferase reporter vectors containing WT or Mut-HK2 3′ UTR with control or miR-125b-5p into HT-29 and SW620 cells. We observed transfection of miR-125b-5p led to a significantly decreased luciferase activity of the luciferase reporter containing WT 3′ UTR of HK2, but not the mutant reporter (Figures 5C,D). The negative correlation between miR-125b-5p and HK2 mRNA was further verified in colon tumors that higher HK2 mRNA expressions accompanied lower miR-125b-5p (Figure 5E). These data confirmed that miR-125b-5p could directly target HK2 in colon cancer.

**Figure 6:**
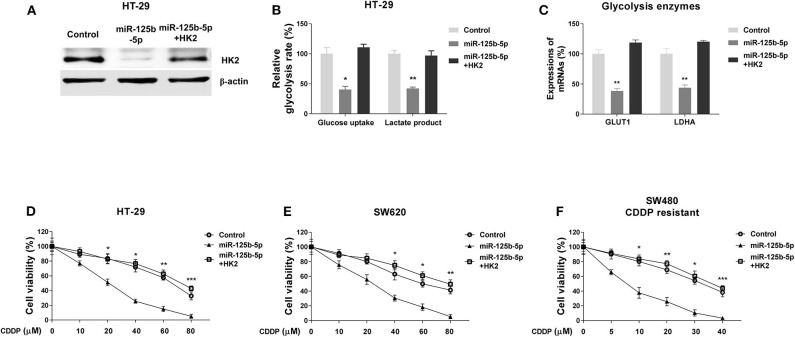
Restoration of HK2 in miR-125b-5p–overexpressing cells recovers glycolysis and cisplatin resistance. **(A)** HT-29 cells were transfected with control miR, miR-125b-5p alone, or miR-125b-5p plus HK2; the expressions of HK2 were measured by Western blot. β-Actin was an internal control. **(B)** The glucose uptake and lactate product assays were performed from the above cells. **(C)** The mRNA expressions of GLUT1 and LDHA were measured from the above cells. **(D)** HT-29, **(E)** SW620, and **(F)** SW480/CDDP R cells were transfected with control miR, miR-125b-5p alone, or miR-125b-5p plus HK2; cells were treated with cisplatin at the indicated concentrations. Cell viability was measured by MTT assay. Data are presented as mean ± SD. *p < 0.05, **p < 0.01, and ***p < 0.001.

To investigate whether the miR-125b-5p–regulated glycolysis inhibition and cisplatin sensitivity were through targeting HK2, we designed rescue experiments by transfecting HT-29 cells with control miRNAs, miR-125b-5p alone, or miR-125b-5p with HK2 overexpression vector. The restoration of HK2 protein was confirmed by Western blot results (Figure 6A). Moreover, cotransfection of miR-125b-5p and HK2 successfully restored the miR-125b-5p–suppressed glucose uptake and lactate product (Figure 6B). Expectedly, HT-29 cells with HK2 restoration showed significantly increased mRNA expressions of GLUT1 and LDHA, other glycolysis key enzymes to normal levels (Figure 6C). We further assessed whether HK2 recovery could desensitize the miR-125b-5p–overexpressing colon cancer cells to cisplatin. Subsequently, HT-29 and SW620 cells without or with HK2 restoration, as well as control and miR-125b-5p–overexpressing cells, were treated with increased concentrations of cisplatin. Results in Figures 6D,E showed cells with HK2 recovery were less sensitive to cisplatin compared with miR-125b-5p–overexpressing cells. We observed SW480/CDDP R cells displayed increased sensitivity to cisplatin with miR-125b-5p overexpression; however, such sensitization was overridden by HK2 rescue (Figure 6F). Taken together, these rescue results verified the miR-125b-5p–suppressed glycolysis and CDDP resistance were through directly targeting HK2 in colon cancer cells.

### Blocking DANCR Increases the Cisplatin Sensitivity of Colon Cancer Cells Through Promoting the miR-125b-5p/HK2 Axis *in vitro* and *in vivo*

**Figure 7:**
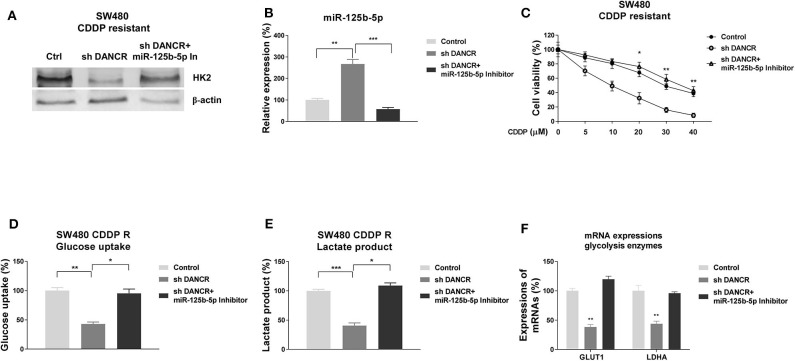
DANCR promotes cisplatin resistance via miR-125b-5p/HK2 axis. **(A)** SW480/CDDP R cells were transfected with control shRNA, DANCR shRNA alone, or sh DANCR plus miR-125b-5p antisense; the expressions of HK2 and **(B)** miR-125b-5p were measured by Western blot or qRT-PCR, respectively. β-Actin was an internal control. **(C)** SW480/CDDP R cells were transfected with control shRNA, DANCR shRNA alone, or sh DANCR plus miR-125b-5p antisense; cells were treated with cisplatin at the indicated concentrations. Cell viability was measured by MTT assay. **(D)** SW480/CDDP R cells were transfected with control shRNA, DANCR shRNA alone, or sh DANCR plus miR-125b-5p antisense; the glucose uptake, **(E)** lactate product, and **(F)** glycolysis enzymes were measured. Data are presented as mean ± SD. *p < 0.05, **p < 0.01, and ***p < 0.001.

Given that DANCR and miR-125b-5p/HK2 inversely regulate cisplatin sensitivity of colon cancer, we examined whether DANCR influenced cisplatin resistance through suppressing the miR-125b-5p/HK2 axis. Thus, SW480/CDDP R cells were transfected with control, DANCR shRNA, or DANCR shRNA plus miR-125b-5p–antisense. Cells with DANCR knocked down displayed significantly downregulated HK2 (Figure 7A). However, such suppression was overcome by further miR-125b-5p inhibition (Figure 7A), suggesting the DANCR-regulated HK2 expression was through inhibiting miR-125b-5p. As we expected, miR-125b-5p levels were induced by DANCR inhibition and further suppressed by anti–miR-125b-5p (Figure 7B). SW480/CDDP R cells with cotransfection of DANCR shRNA plus anti–miR-125b-5p apparently recovered cisplatin resistance (Figure 7C). To further validate whether the DANCR-regulated HK2 expression could affect the glycolysis rate of colon cancer cells, glucose uptake, and lactate product and glycolysis enzyme expressions were measured in SW480/CDDP R cells with DANCR inhibition alone or DANCR knockdown plus miR-125b-5p inhibition. Results in Figures 7D–F consistently demonstrated DANCR knockdown plus miR-125b-5p inhibition successfully recovered glycolysis rate of cisplatin-resistant cells.

**Figure 8:**
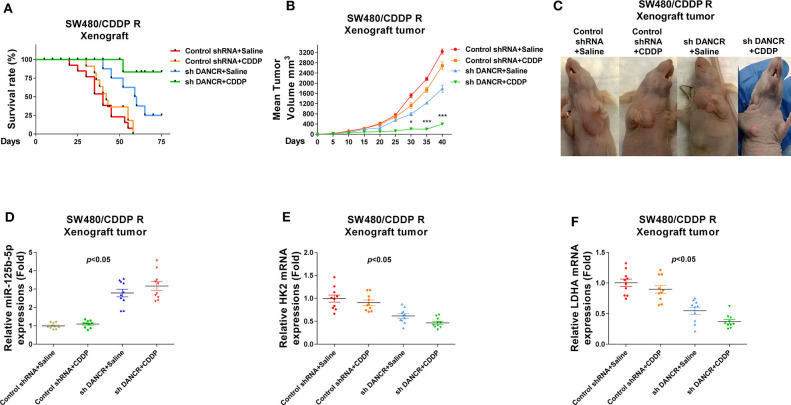
Effects of silencing DANCR on cisplatin resistance of colon cancer *in vivo*. **(A)** SW480/CDDP R cells stably transfected with control shRNA or sh DANCR were subcutaneously injected into BALB/c mice (*n* = 10). Mice were continually treated with cisplatin or control saline for 2 months. The survival rates of mice were recorded and analyzed. **(B)** Tumor volumes from the above treated mice were measured each 5 days. **(C)** Mice from the above treatments were sacrificed, and the tumors from each group were visualized. **(D)** Expressions of miR-125b-5p, **(E)** HK2 mRNAs, and **(F)** LDHA mRNAs in tumors from the above treated mice. Data are presented as mean ± SD. *p < 0.05, and ***p < 0.001.

Finally, we verified the above proposed mechanisms by which DANCR promoted cisplatin resistance using an *in vivo* xenograft model. SW480/CDDP R cells stably infected with control shRNA or shDANCR were subcutaneously injected into the right flank of 6-week-old female BALB/c nude mice for establishing xenograft tumors. Mice were treated with cisplatin or control saline via i.p. injection twice a week (Figure 8A). Most of control shRNA-infected mice that received cisplatin treatment died within 2 months. Compared with mice that received only DANCR knockdown but saline treatment, the combination of DNACR knockdown and cisplatin achieved a significantly prolonged survival rate (Figure 8A), suggesting an effective antidrug resistance due to DANCR inhibition. After 60 days, the mice were sacrificed, and the tumors were collected (Figures 8B,C). Consistent results demonstrated that mice with DANCR knockdown had longer survival potential under cisplatin treatments. Moreover, qRT-PCR results from the collected mice tumors showed the mRNA expressions of HK2 and LDHA were suppressed, but miR-125b-5p levels were induced in xenograft tumors with DANCR knockdown (Figures 8D–F). In summary, the above results revealed that tumors generated from SW480/CDDP R cells with DANCR knockdown were more sensitive to cisplatin treatment through miR-125b-5p upregulation and glycolysis inhibition.

## Discussion

Colon cancer is the third most common type of malignancy and one of the major causes of cancer-associated mortality (1, 2). Seriously, the development of drug resistance established an obstacle in the treatment of advanced colon cancer. Cisplatin exerts its anticancer effects via multiple mechanisms such as creating DNA lesions to activate the DNA damage response, leading to the induction of cancer cell apoptosis (4). Although an impressive initial response, a large fraction of colon cancer patients develops cisplatin resistance, leading to chemotherapeutic failure. An intense research has reported that LncRNA-DANCR is involved in chemoresistance. DANCR was known to mediate cisplatin resistance in glioma cells via activating AXL/PI3K/Akt/NF-κB signaling pathway (32). In addition, DANCR has been reported to regulate paclitaxel sensitivity in prostate cancer *via* sponging miR-135a (15). These studies suggest DANCR might play important roles in chemoresistance of colon cancer. In this study, we observed that DANCR was significantly upregulated in colon cancer tissues and cells, consistent with previous reports (12). Moreover, by establishing cisplatin-resistant colon cancer cells, we found DANCR was clearly upregulated in CDDP resistant cells. Silencing DANCR effectively resensitized CDDP-resistant colon cancer cells. These discoveries indicated lncRNA-DANCR is a potential marker for screening cisplatin-resistant colon cancer.

We next investigated the molecular mechanisms for the DANCR-mediated cisplatin resistance. Accumulating evidence demonstrated that miRNAs are a class of essential regulators of cancers and take charge of diverse tumors processes (16). Furthermore, recent studies revealed that lncRNA could directly bind to miRNAs by acting as competing endogenous RNAs (18–20). The inhibited target miRNAs of lncRNAs will further result in derepressing of miRNA targets (18). Previous studies indicated multiple miRNA targets of DANCR, such as miR-135a (15), miR-27a (33), and miR-149 (22). However, the direct miRNA targets of DANCR and the DANCR/miRNA interaction in mediating cisplatin resistance of colon cancer have not been elucidated. Based on the bioinformatics analysis through starBase of ENCORI and experimental results from Western blot and luciferase assay, we first demonstrated that DANCR functioned as a ceRNA of miR-125b-5p, resulting in modulating the expressions of HK2, a direct target of miR-125b-5p. Given the consequent results that miR-125b-5p was apparently negatively associated with cisplatin resistance in colon cancer, both our *in vitro* and *in vivo* data consistently elucidated a DANCR–miR-125b-5p/HK2 axis in the acquired cisplatin resistance, suggesting targeting this molecular pathway contributes to develop therapeutic approaches against chemoresistance of colon cancer.

A new metabolic hallmark of cancer has been proposed and investigated recently (24, 25). The metabolic feature of cancer cell is characterized by preferential dependence on anaerobic glycolysis (the process of converting glucose into pyruvate and further into lactate) for energy production and building block supply for proliferation even under an oxygen-sufficient environment (23–25). Furthermore, growing evidence revealed that elevated glycolysis rate was positively associated with chemoresistance (27, 29). Intriguingly, it has been recognized that tumor glycolysis is a potential target for developing anticancer agents. Hexokinase 2, a key glycolytic enzyme that phosphorylates glucose into G6P, has been reported to be upregulated in diverse cancers and strongly linked to chemoresistance (34). For instance, in ovarian cancer, HK2 is highly expressed in cancer tissues compared with normal, benign, and borderline ovarian tumors (35), suggesting targeting HK2 is a promising approach for cancer treatment. In this study, we identified HK2 as a direct target of miR-125b-5p in colon cancer. Moreover, the inverse correlation was validated in colon cancer tissues. Although miR-125b-5p targeting HK2 has been reported (36, 37), our results integrated the miR-125b-5p/HK2-glycolysis pathway into the DANCR-promoted cisplatin resistance via both *in vitro* and *in vivo* models.

## Conclusions

In summary, our results elucidated an oncogenic role of lncRNA-DANCR in the cisplatin resistance of colon cancer. We proposed DANCR specifically sponges miR-125b-5p to derepress the expression of HK2, a direct target of miR-125b-5p in colon cancer, presenting the DANCR/miR-125b-5p/HK2-glycolysis axis as a new therapeutic target for overcoming cisplatin resistance of colon cancer.

## Data Availability Statement

The original contributions presented in the study are included in the article/supplementary materials, further inquiries can be directed to the corresponding author/s.

## Ethics Statement

The studies involving human participants were reviewed and approved by Institutional Ethical Review Boards of the Affiliated Cancer Hospital & Institute of Guangzhou Medical University. The patients/participants provided their written informed consent to participate in this study. The animal study was reviewed and approved by Institutional Ethical Review Boards of the Affiliated Cancer Hospital & Institute of Guangzhou Medical University.

## Author Contributions

HS and KL designed the study. JF and GL carried out the experiments. XZ analyzed and interpreted the results. YF performed the statistical analysis. XZ wrote and edited the manuscript. All authors contributed to the article and approved the submitted version.

## Conflict of Interest

The authors declare that the research was conducted in the absence of any commercial or financial relationships that could be construed as a potential conflict of interest.

## Supplementary Material

Figure S1 Bioinformatics analysis of the expressions of DANCR in colon cancer tissues and normal colon tissues from starBase.

Figure S2 Bioinformatics analysis of the expressions of miR-125b-5p in colon cancer tissues and normal colon tissues from starBase.

Figure S3 Bioinformatics analysis of the correlation between miR-125b-5p and DANCR in colon cancer tissues from starBase.

Figure S4 Bioinformatics analysis of the correlation between miR-125b-5p and HK2 mRNA in colon cancer tissues from starBase.
