# Supplementary figures and images for: LncRNA-DANCR Interferes With miR-125b-5p/HK2 Axis to Desensitize Colon Cancer Cells to Cisplatin vis Activating Anaerobic Glycolysis
Source: Front Oncol. 2020 Jul 17;10:1034. doi: 10.3389/fonc.2020.01034 (PMC7379395; doi:10.3389/fonc.2020.01034)

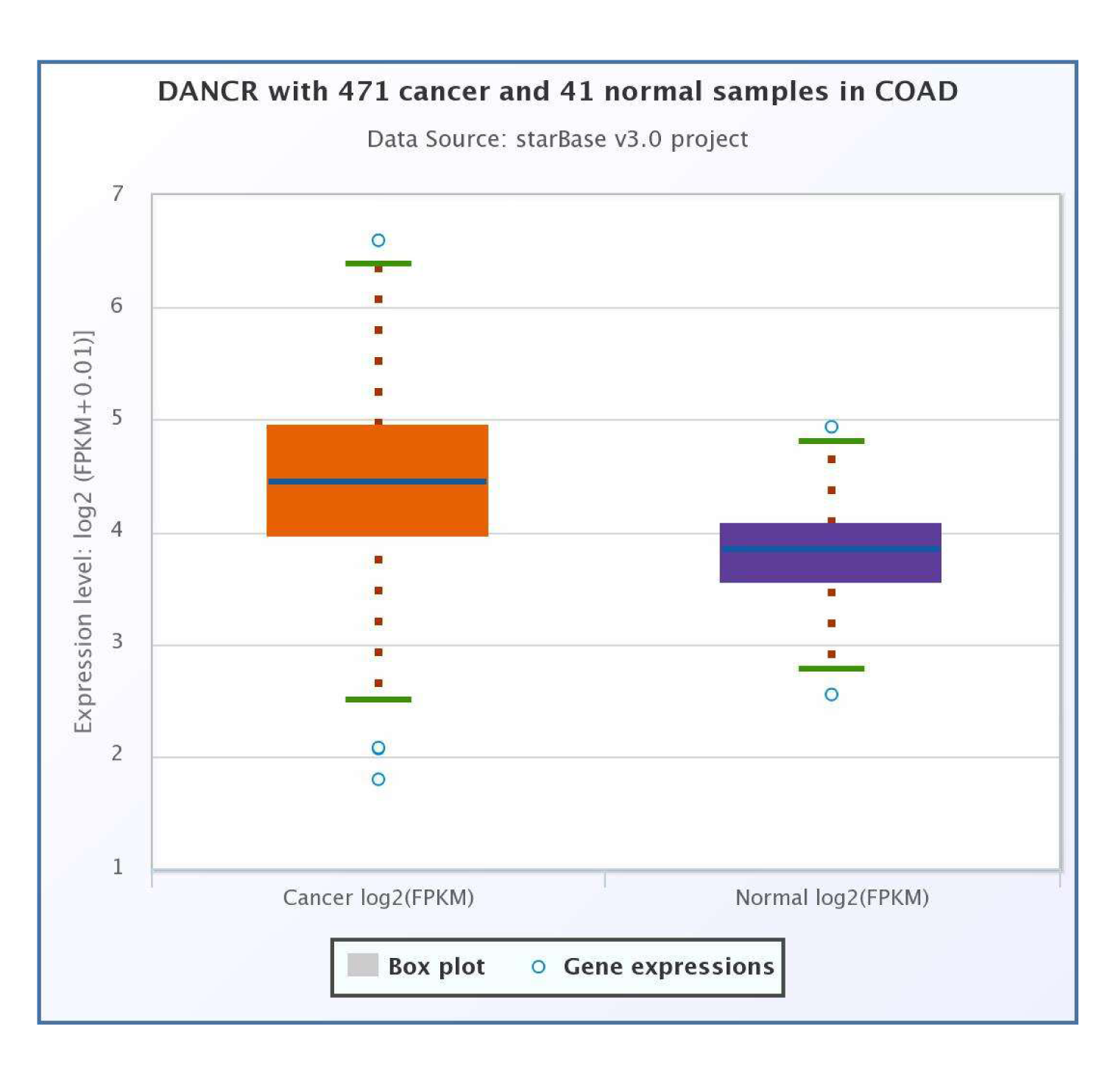

Supplement: Figure S1 — Bioinformatics analysis of the expressions of DANCR in colon cancer tissues and normal colon tissues from starBase. [file Image_1.tif]

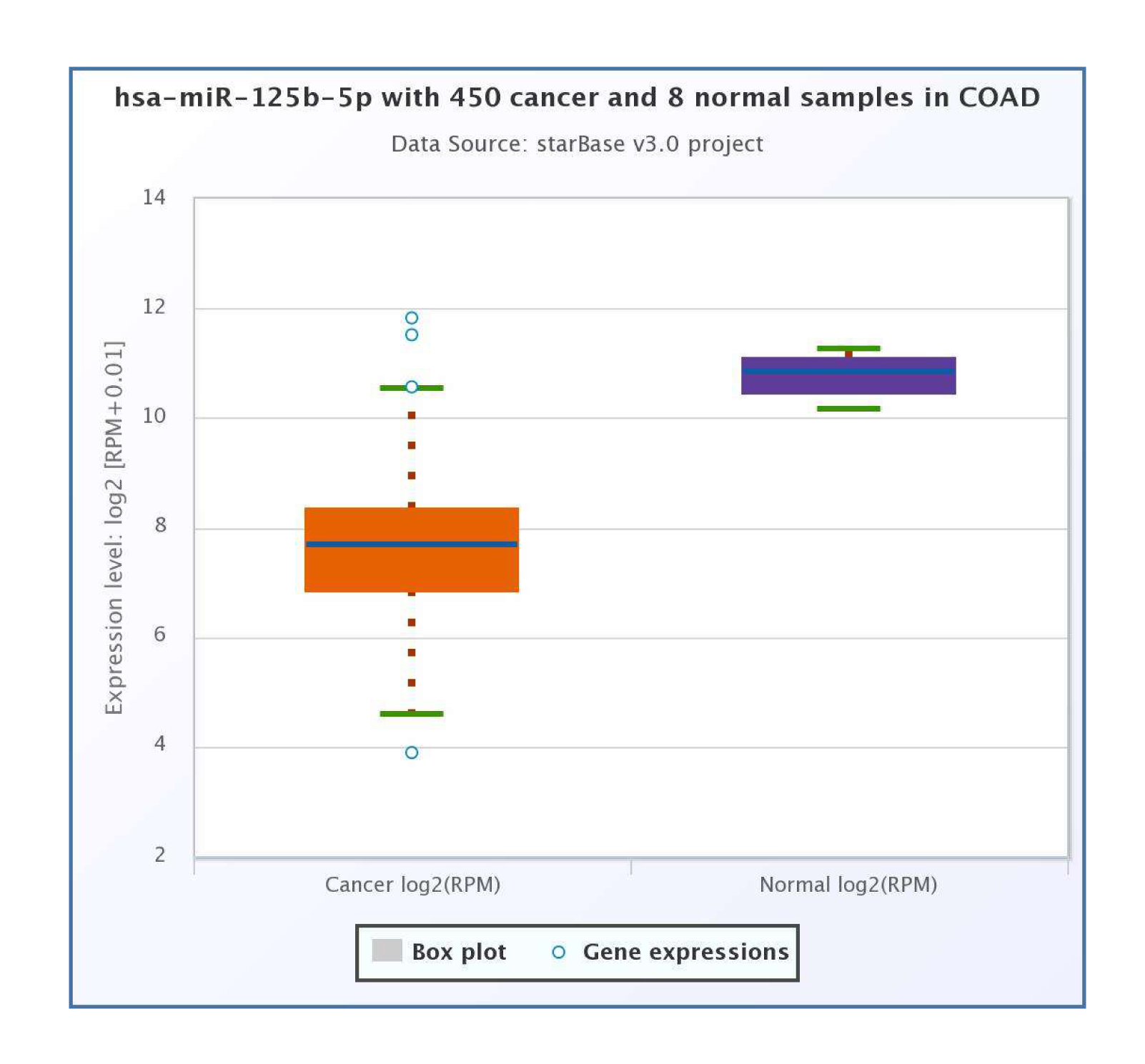

Supplement: Figure S2 — Bioinformatics analysis of the expressions of miR-125b-5p in colon cancer tissues and normal colon tissues from starBase. [file Image_2.tif]

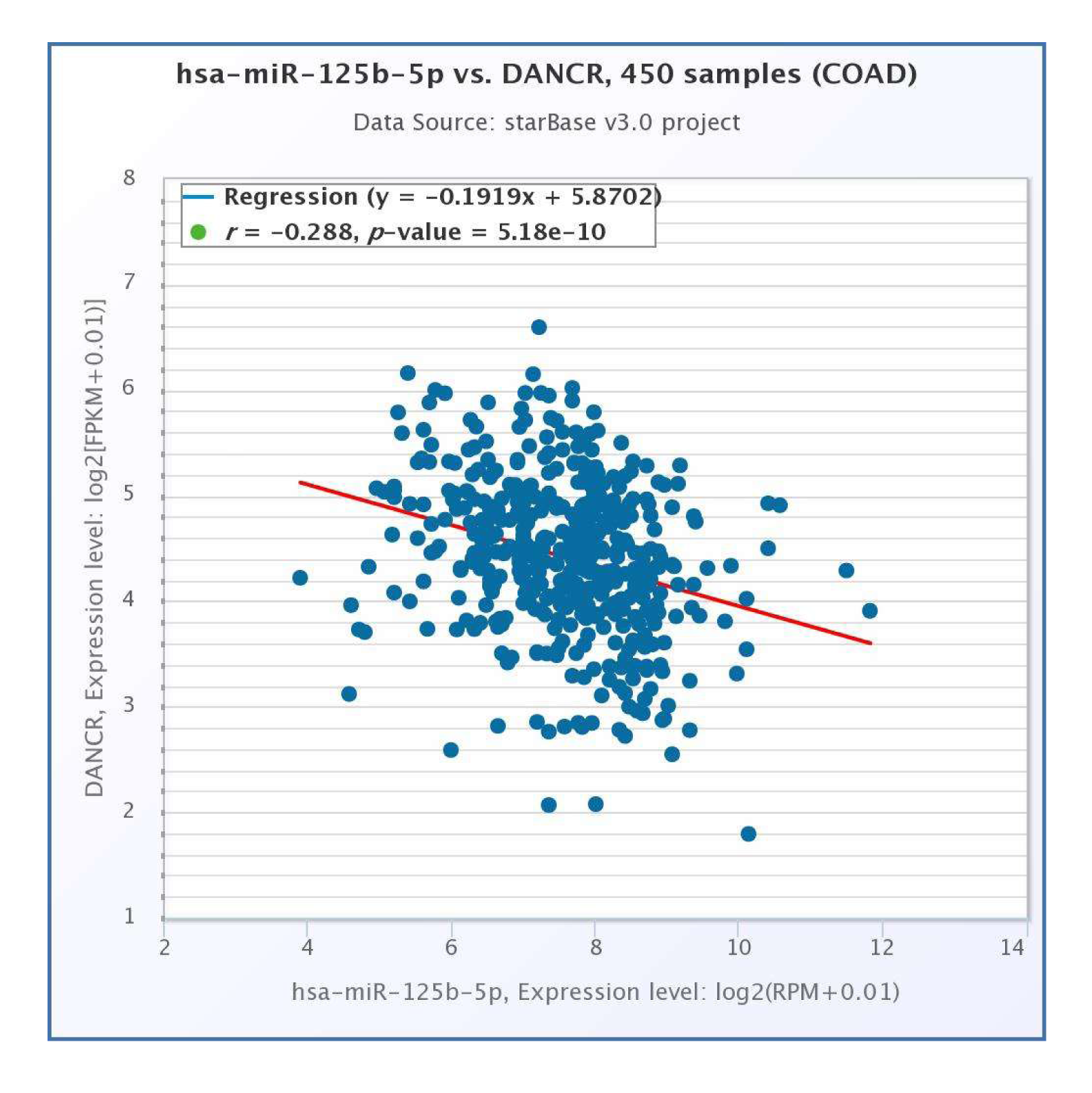

Supplement: Figure S3 — Bioinformatics analysis of the correlation between miR-125b-5p and DANCR in colon cancer tissues from starBase. [file Image_3.tif]

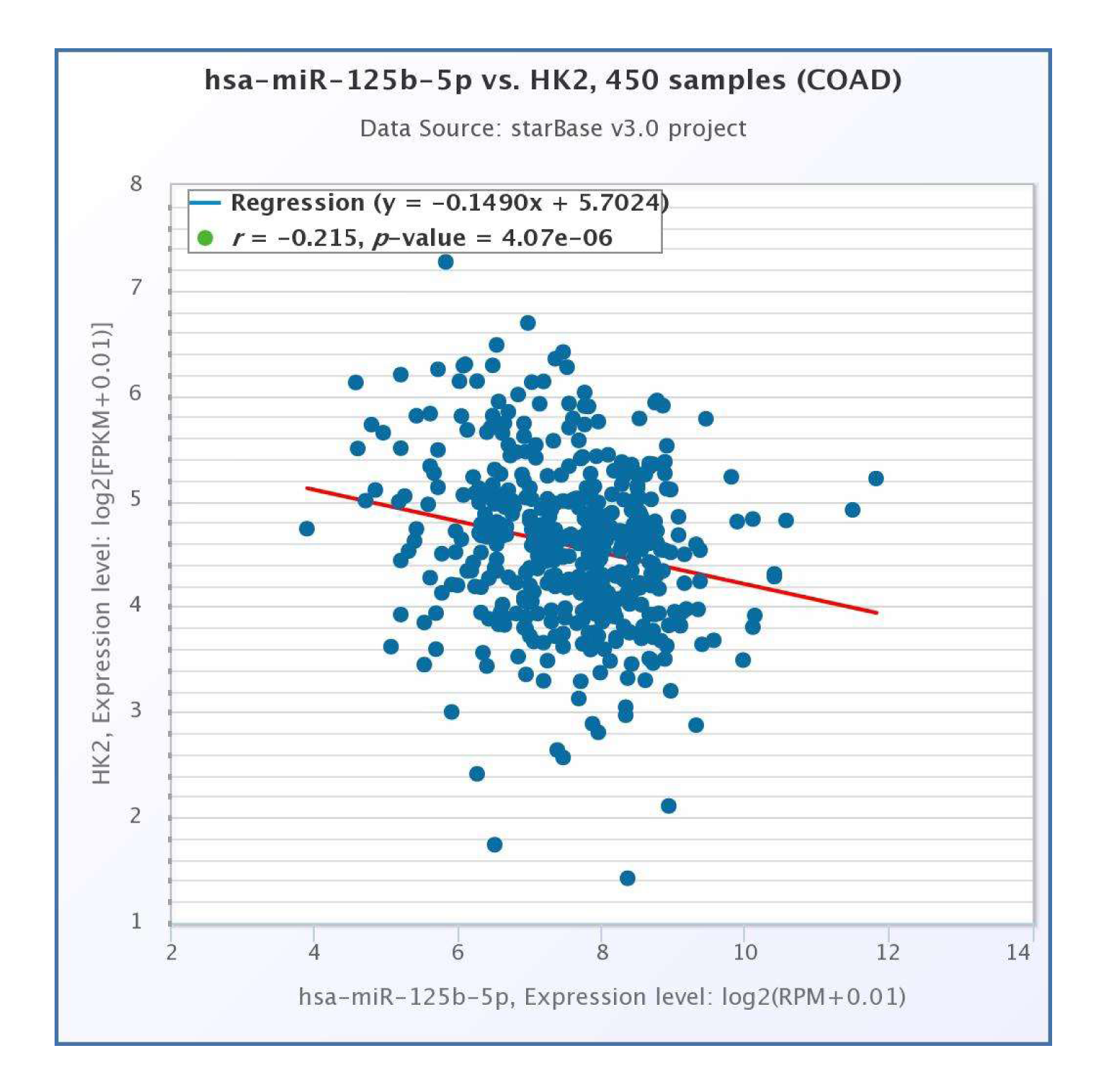

Supplement: Figure S4 — Bioinformatics analysis of the correlation between miR-125b-5p and HK2 mRNA in colon cancer tissues from starBase. [file Image_4.tif]
